# High‐risks drug adverse events associated with Cetirizine and Loratadine for the treatment of allergic diseases: A retrospective pharmacovigilance study based on the FDA adverse event reporting system database

**DOI:** 10.1002/clt2.12392

**Published:** 2024-09-10

**Authors:** Weili Kong, Yijun Dong, Sixi Yi, Wei Mo, Hui Yang

**Affiliations:** ^1^ Department of Otolaryngology‐Head & Neck Surgery West China Hospital Sichuan University Chengdu Sichuan China

**Keywords:** adverse drug event, antihistamine, FAERS, real‐world data analysis, safety signal

## Abstract

**Background:**

Cetirizine and Loratadine are the two best‐selling second‐generation antihistamines for allergic diseases. This study aims to provide a comparative analysis of the differences in adverse drug events (ADEs) between these two medications, which can assist clinicians in making appropriate treatment decisions.

**Methods:**

ADE reports related to Cetirizine and Loratadine obtained from the FDA adverse event reporting system (FAERS) database were analyzed using disproportionality analysis and Bayesian analysis to evaluate and compare the ADE signals of both drugs.

**Results:**

A total of 28,051 and 28,073 ADE reports were retrieved from the FAERS database related to Cetirizine and Loratadine, respectively, with both drugs showing a predominance of middle‐aged females. Specifically, Loratadine was associated with respiratory symptoms, mainly nasal symptoms such as rhinorrhea (*n* = 326, ROR 6.75), sneezing (*n* = 251, ROR 15.24), and nasal congestion (*n* = 185, ROR 4.25), while Cetirizine did not show this association. Notably, both drugs exhibited strong signals for somnolence in the nervous and psychiatric systems, especially Cetirizine (Cetirizine, *n* = 2556, ROR 10.52 vs. Loratadine, *n* = 1200, ROR 7.76). Additionally, Cetirizine itself showed strong signals for attention disturbance (*n* = 233, ROR 3.3), while Loratadine was associated with nervousness (*n* = 145, ROR 3.3). Further exploration revealed more severe adverse reactions closely associated with Cetirizine, including hallucinations, aggression, and abnormal behavior. Importantly, Cetirizine was significantly associated with the occurrence of pericarditis (*n* = 138, ROR 8.13), potentially leading to serious adverse consequences.

**Conclusion:**

Compared to Loratadine, Cetirizine poses a greater potential risk in the nervous and psychiatric systems. Additionally, this study reveals previously underestimated potential cardiac toxicity of Cetirizine; albeit at a relatively low incidence rate, the high signal intensity warrants further attention and exploration. These findings highlight the need for enhanced patient monitoring and therapy optimization when prescribing these medications, ensuring better management of allergic diseases while minimizing risks.

## 1 INTRODUCTION

Histamine, synthesized from histidine by histidine decarboxylase, is primarily stored in mast cells and basophilic granulocytes and plays a pivotal role in regulating allergic and inflammatory responses.^1^ Upon exposure to allergens, histamine is induced to release from cells and subsequently binds to H1 receptors on target cells such as smooth muscle cells, endothelial cells, and sensory nerve terminals,^2^, ^3^ promoting vasodilation, bronchoconstriction, increased vascular permeability, and sensory nerve stimulation, leading to allergic symptoms such as itching, sneezing, nasal congestion, and bronchial constriction.^4^, ^5^

In response to histamine‐mediated allergic reactions, the development of antihistamine medications has been a significant focus of pharmacological research. First‐generation antihistamines, such as diphenhydramine and chlorpheniramine, were initially used to treat allergic conditions. However, its efficacy was often limited by sedation and anticholinergic side effects, prompting the development of second‐generation antihistamines.^3^, ^6^ Second‐generation antihistamines, including cetirizine, Loratadine, and fexofenadine, address the drawbacks of their predecessors by enhancing selectivity for H1 receptors and minimizing penetration of the blood‐brain barrier, thereby minimizing central nervous system side effects.^7^ These medications offer enhanced efficacy in relieving allergic symptoms while maintaining good safety profiles, making them preferred options in the clinical management of allergic diseases.

Cetirizine^8^ and Loratadine,^9^ among the most widely used second‐generation antihistamines, are primarily indicated for allergic diseases of the respiratory system and skin, including chronic idiopathic urticaria, perennial allergic rhinitis, allergic rhinitis, allergic conjunctivitis, and asthma.^10^, ^11^, ^12^ However, the safety of these drugs is equally important as their efficacy. Cetirizine and Loratadine have both been approved by the food and drug administration (FDA) as over‐the‐counter (OTC) medications in the United States, allowing patients easier access to these drugs for treating allergy symptoms.^13^ Specifically, Cetirizine received OTC approval in 2002, while Loratadine was approved in 2003. This change not only enhanced the convenience of medication for patients but also promoted the widespread use of these two drugs.^14^ Yet, comprehensive reports on adverse events(AEs) associated with these two medications are currently lacking, and monitoring and synthesis of AE reports related to antihistamine use remain relatively underestimated. Moreover, there is also a lack of guiding evidence for the selection of these two second‐generation antihistamines in practical clinical scenarios, highlighting the need for safety comparisons between them.

The FDA adverse event reporting system (FAERS), established in 1969, is a comprehensive database managed by the U.S. Food and Drug Administration (FDA). By collecting comprehensive reports of AEs, medication errors, and product quality issues voluntarily submitted by healthcare professionals, consumers, and manufacturers, information gathered from FAERS plays a crucial role in identifying potential drug safety issues, monitoring trends in AE reporting, detecting emerging risks, and informing healthcare providers, patients, and policymakers about the risks and benefits associated with specific drugs and medical products.^15^ It provides a solid foundation for monitoring and comparing adverse drug reactions.

Here, this study sought to evaluate and characterize the safety profiles of Cetirizine and Loratadine using the FAERS database with the goal of understanding the differences in efficacy and toxicity between these second‐generation antihistamines. Secondarily, the study will compare the two profiles to provide concrete evidence to assist in the appropriate choice and management of these medications in real clinical contexts.

## 2 METHODS

### 2.1 Data source

This study selected American Standard Code for Information Interchange (ASCII) report files from the FAERS database (https://www.fda.gov/drugs/fda‐adverse‐event‐reporting‐system‐faers‐public‐dashboard) from the first quarter of 2004 to the fourth quarter of 2023. The data were imported and processed using R (version 4.3.1).

### 2.2 Data extraction

**FIGURE 1:**
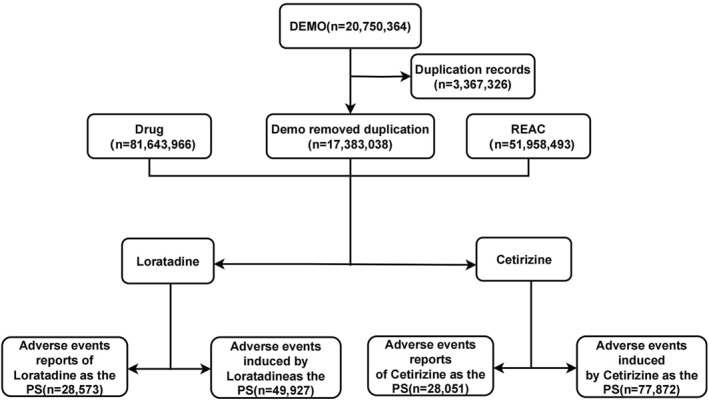
The flow diagram of selecting Cetirizine‐related and Loratadine‐related ADEs from FAERS database. ADEs, adverse drug events; FAERS, FDA adverse event reporting system.

Following previous studies, duplicate reports were removed. For data in the DEMO table with the same conditions, only the latest report based on that date was retained. The screening process for adverse drug events (ADEs) related to both drugs is illustrated in Figure 1. The drug names were standardized using the Medex_Unstructured Information Management Architecture_1.8.3 system. Reports related to Cetirizine excluded those using Levocetirizine, or containing ephedrine or other drug components (exclusion criteria: ‘!∼levocetirizine|!∼reactine|!∼pseudoephedrine hydrochloride|!∼montelukast|!∼linzess’). And reports related to Loratadine excluded those using DesLoratadine or containing ephedrine or other drug components (exclusion criteria: ‘!∼levocetirizine|!∼reactine|!∼pseudoephedrine hydrochloride|!∼montelukast|!∼linzess’).

Each ADE report was encoded using the preferred terms (PT) from the medical dictionary for regulatory activities (MedDRA v25.0) along with its corresponding system organ class (SOC). Patient gender, age at ADE onset, weight, reporting year, reporting country, reporter, outcome, administration route, and other parameters were included in this study. Additionally, indications and concomitant medications associated with ADEs were also extracted.

### 2.3 Data analysis

Each analysis of the association between drug exposure and ADEs (signals) was based on a 2 × 2 contingency table using the frequency‐based method of disproportionality analysis (DPA). The reporting odds ratio (ROR)^16^ was used to measure the association between the observed frequency in the exposed population and the non‐exposed population. Higher ROR indicates a stronger statistical relationship between the target drug and the target ADE. To minimize false‐positive AE signals, we employed the Bayesian Confidence Propagation Neural Network (BCPNN), a Bayesian method that does not rely on frequentist statistics to validate the AE signals under consideration. A signal was deemed significant if the following criteria were met: the value of a was ≥3, the lower threshold of the ROR 95% Confidence Interval (CI) exceeded 1, the proportional reporting ratio was greater than 2 with a corresponding *χ* ^2^ value exceeding 4, and the lower limit of the IC 95% CI was greater than 0. All analyses were conducted and illustrated using R (version 4.3.1). Categorical data were presented as *n* (%) and Breslow‐Day statistics were used to compare the ROR of ADEs between Cetirizine and Loratadine. The relevant data analysis R code can be obtained from the attachment.

## 3 RESULTS

### 3.1 Description analysis

**FIGURE 2:**
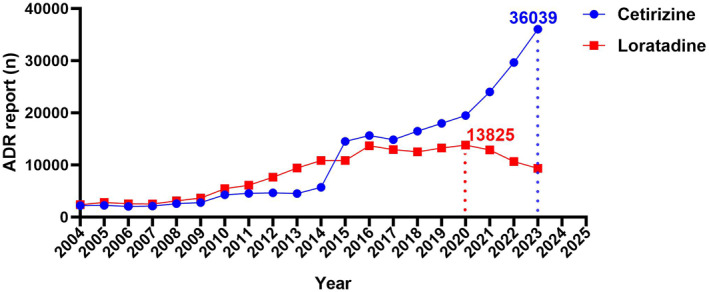
The number of Cetirizine‐related ADEs and Loratadine‐related ADEs reported yearly after 2004. The blue line represents the reports of Cetirizine while the red line represents the reports of Loratadine. *X*‐axis shows the timeline when the drug was used, and *Y*‐axis displays the number of reports per year. ADEs, adverse drug events.

From the first quarter of 2004 to the fourth quarter of 2023, the FAERS database collected a total of 20,750,364 related ADE reports, of which 28,051 were related to Cetirizine and 28,573 were related to Loratadine (the flow charts were shown in Figure 1). Since their inclusion in 2004, reports of ADEs related to Cetirizine and Loratadine have shown a steady increasing trend until a notable inflection point in 2020. Since then, reports of ADEs related to Cetirizine have increased more rapidly, reaching the highest number of reports in 2023 at 36,039 cases. Conversely, reports of ADEs related to Loratadine reached their peak in 2020 at 13,825 cases and have since shown a relatively stable or even slightly declining trend (Figure 2 and Table S1).

Furthermore, we compared other basic characteristics of Cetirizine and Loratadine‐related ADEs (Table 1). Both drugs are typically administered orally, and oral administration was predominant in ADE reports (Cetirizine vs. Loratadine, 54.1% vs. 71.64%). The average ages in the ADE reports for both drugs were similar (Mean age, Cetirizine vs. Loratadine, 46 vs. 44 years old), and the majority of reports came from females (Cetirizine vs. Loratadine, 50.8% vs. 53.62%). Given that the FAERS database is supported by the American FDA, the vast majority of ADE reports came from the United States, particularly for Loratadine (Cetirizine vs. Loratadine, 79.74% vs. 94.95%). Interestingly, consumers themselves seem to be more concerned about the occurrence of AEs (76.9% of Cetirizine‐related ADE reports and 89.83% of Loratadine‐related ADE reports were from consumers), with only about 10% of reports coming from healthcare professionals. Additionally, besides unknown severe factors, hospitalization, disability, life‐threatening conditions, and death might be serious consequences associated with the use of these two drugs.

**TABLE 1:** Characteristics of reports associated with Cetirizine and Loratadine from Q1 of 2004 to Q4 of 2023.

Variables	Cetirizine (*n*, %) *N* = 28,051	Loratadine (*n*, %) *N* = 28,573
Age (median (Q1, Q3))	46.00 (22.00, 67.00)	44.00 (8.00, 69.00)
Sex		
Female	14,250 (50.80)	15,322 (53.62)
Male	7505 (26.75)	9270 (32.44)
Unknown	6296 (22.44)	3981 (13.93)
Weight (median (Q1, Q3))	68.04 (55.00, 82.00)	66.00 (52.00, 81.63)
Reporter		
Consumer	21,571 (76.90)	25,667 (89.83)
Physician	2080 (7.42)	719 (2.52)
Other health‐professional	1794 (6.40)	1245 (4.36)
Pharmacist	1700 (6.06)	524 (1.83)
Lawyer	15 (0.05)	9 (0.03)
Registered nurse	1 (0.00)	1 (0.00)
Unknown	890 (3.17)	408 (1.43)
Outcomes		
Other serious	6735 (65.44)	2418 (62.02)
Hospitalization	2057 (19.99)	983 (25.21)
Disability	468 (4.55)	96 (2.46)
Life threatening	468 (4.55)	162 (4.15)
Death	337 (3.27)	177 (4.54)
Congenital anomaly	189 (1.84)	22 (0.56)
Permanent impairment/damage	38 (0.37)	41 (1.05)
Reported countries		
United States	20,218 (79.74)	25,359 (94.49)
United Kingdom	1957 (7.72)	549 (2.05)
France	674 (2.66)	51 (0.19)
Germany	417 (1.64)	NA
Italy	374 (1.47)	NA
Canada	371 (1.46)	102 (0.38)
Netherlands	187 (0.74)	86 (0.32)
Spain	114 (0.45)	NA
Singapore	104 (0.41)	NA
Turkey	84 (0.33)	NA
Japan	75 (0.30)	77 (0.29)
India	71 (0.28)	NA
Belgium	67 (0.26)	NA
Russia	52 (0.21)	NA
Australia	51 (0.20)	214 (0.80)
China	NA	114 (0.42)
Others	540 (2.13)	287 (1.07)
Route	NA	NA
Oral	15,161 (54.10)	20,458 (71.64)
Transplacental	190 (0.68)	24 (0.08)
Topical	13 (0.05)	NA
Ophthalmic	NA	17 (0.06)
Others	12,659 (45.17)	8057 (28.21)

Abbreviation: NA, not applicable.

In addition, the top five indications and accompanying medications in ADE reports for each drug were identified, as shown in Table 2. Clearly, Cetirizine and Loratadine were commonly used to treat allergies (including drug, food, plant, animal, and mixed allergies) and hypersensitivity (Cetirizine vs. Loratadine, 35.67% vs. 46.94%). However, Cetirizine‐related ADE reports seemed to be more commonly associated with allergies (20.95%), while Loratadine was more commonly used for hypersensitivity (30.2%). Additionally, both drugs are commonly used for allergic skin conditions, allergic rhinitis and asthma.

**TABLE 2:** Top five indications and top five concomitant drugs in ADE reports of Cetirizine and Loratadine.

	Cetirizine	*n* (%)	Loratadine	*n* (%)
Indications	Allergy	4078 (20.95)	Hypersensitivity	6761 (30.2)
	Hypersensitivity	2865 (14.72)	Allergy	3747 (16.74)
	Allergic skin	1874 (9.63)	Allergic rhinitis	2123 (9.48)
	Allergic rhinitis	1390 (7.14)	Allergic skin	976 (4.36)
	Asthma	138 (0.71)	Asthma	324 (1.45)
Concomitant medication	Levothyroxine sodium	462 (24.72)	Calcium carbonate	346 (40.52)
	Sulfasalazine	395 (21.13)	Aspirin	171 (20.02)
	Methotrexate	393 (21.03)	Synthroid	147 (17.21)
	Simvastatin	313 (16.75)	Fish oil	114 (13.35)
	Lansoprazole	306 (16.37)	Magnesium	76 (8.90)

Abbreviation: ADE, adverse drug event.

In most of the Cetirizine‐related ADE reports, the most commonly reported concomitant medications were Levothyroxine sodium (24.72%), Sulfasalazine (21.13%), Methotrexate (21.03%), Simvastatin (16.75%), and Lansoprazole (16.37%), with similar proportions among these top five drugs. However, for Loratadine, Calcium carbonate apparently topped the list at 40.52%, followed by Aspirin at 20.02%, Synthroid (17.21%), Fish oil (13.35%), and Magnesium (8.90%).

### 3.2 Disproportionality analysis

Under Cetirizine and Loratadine, strong signal PTs with ROR>1 were identified, with the TOP 30 displayed in Table 3. The differences between the two drugs led to variations in the occurrence rates of ADEs across different SOC and PT categories. Notably, in the general disorders and administration site conditions, there were numerous reports of drug ineffectiveness or unstable efficacy for both drugs. Comparatively, Loratadine had more reports of drug ineffectiveness than Cetirizine (8685 vs. 4805) and exhibited a higher correlation (ROR 10.24 vs. 3.06). Conversely, Cetirizine had a more pronounced likelihood of unexpected therapeutic response (Cetirizine, *n* = 1482, ROR 26.81 vs. Loratadine, *n* = 451, ROR 12.57). Additionally, both antihistamines had similar rates of reports for drug ineffectiveness for unapproved indications or therapeutic product effects incompletely. Notably, withdrawal syndrome was a high signal ADE unique to Cetirizine (*n* = 828, ROR 15.53), whereas a drug effective for unapproved indications was only reported with Loratadine and had a high signal strength (*n* = 216, ROR 13.64).

**TABLE 3:** Comparison of ADE signals between Cetirizine and Loratadine in various SOCs (Top 30).

SOCs/PTs	Cetirizine	ROR (95% CI)	Loratadine	ROR (95% CI)
General disorders and administration site conditions				
Drug ineffective	4805	3.06 (2.97, 3.15)	8685	10.24 (10, 10.48)
Therapeutic response unexpected	1482	26.81 (25.45, 28.26)	451	12.57 (11.45, 13.8)
Withdrawal syndrome	828	15.53 (14.49, 16.64)	NA	NA
Drug ineffective for unapproved indication	618	9.95 (9.19, 10.78)	511	13.16 (12.06, 14.37)
Therapeutic product effect incomplete	360	4.52 (4.07, 5.01)	200	4 (3.48, 4.6)
Drug effective for unapproved indication	NA	NA	216	13.64 (11.92, 15.6)
Nervous system disorders				
Somnolence	2556	10.52 (10.11, 10.95)	1200	7.76 (7.33, 8.22)
Disturbance in attention	233	3.3 (2.9, 3.75)	NA	NA
Injury, poisoning and procedural complications				
Product use in unapproved indication	2112	8.41 (8.05, 8.79)	732	4.56 (4.24, 4.91)
Product use issue	1360	6.27 (5.94, 6.62)	1291	9.61 (9.09, 10.15)
Overdose	1125	3.95 (3.73, 4.19)	1361	7.77 (7.36, 8.2)
Expired product administered	631	15.6 (14.41, 16.88)	1043	42.47 (39.89, 45.21)
Accidental overdose	612	14.61 (13.48, 15.84)	821	31.89 (29.73, 34.2)
Extra dose administered	406	10.94 (9.92, 12.07)	1380	63.47 (60.07, 67.06)
Product administered to patient of inappropriate age	296	54.16 (48.1, 60.98)	NA	NA
Accidental exposure to product by child	NA	NA	1052	242.68 (226.86, 259.6)
Incorrect dose administered	NA	NA	553	3.7 (3.41, 4.03)
Circumstance or information capable of leading to medication error	NA	NA	162	7.21 (6.18, 8.42)
Skin and subcutaneous tissue disorders				
Pruritus	2039	4.9 (4.68, 5.12)	NA	NA
Urticaria	985	4.98 (4.68, 5.31)	NA	NA
Angioedema	285	5.22 (4.65, 5.87)	NA	NA
Eye disorders				
Eye pruritus	180	5.05 (4.36, 5.85)	NA	NA
Lacrimation increased	NA	NA	156	7.2 (6.15, 8.43)
Gastrointestinal disorders				
Lip swelling	155	3.71 (3.17, 4.35)	NA	NA
Cardiac disorders				
Pericarditis	138	8.13 (6.87, 9.62)	NA	NA
Respiratory, thoracic and mediastinal disorders				
Rhinorrhea	NA	NA	326	6.75 (6.05, 7.53)
Sneezing	NA	NA	251	15.24 (13.45, 17.27)
Nasal congestion	NA	NA	185	4.25 (3.68, 4.92)
Psychiatric disorders				
Nervousness	NA	NA	145	3.3 (2.8, 3.89)

Abbreviations: ADE, adverse drug event; CI, confidence interval; NA, not applicable; ROR, reporting odds ratio; SOC, system organ class.

In Nervous system disorders, both drugs exhibited strong signals for somnolence, especially in the case of Cetirizine (Cetirizine, *n* = 2556, ROR 10.52 vs. Loratadine, *n* = 1200, ROR 7.76). Additionally, Cetirizine itself showed some strong signals for disturbance in attention (*n* = 233, ROR 3.3).

Regarding Injury, poisoning and procedural complications, both drugs demonstrated similar levels of association. Notably, Loratadine was more prone to extra dose administered (Cetirizine vs. Loratadine, 406 vs. 1380) with a higher signal strength (ROR, Cetirizine vs. Loratadine, 10.94 vs. 63.47). Moreover, the occurrence of product administered to patients of inappropriate age was only reported with Cetirizine (*n* = 296, ROR 54.16). Conversely, Loratadine had unique ADEs such as accidental exposure to product by child (*n* = 1052, ROR 242.68), incorrect dose administered (*n* = 553, ROR 3.7), and circumstance or information capable of leading to medication error (*n* = 162, ROR 7.21).

In skin and subcutaneous tissue disorders, Cetirizine exhibited strong signals for pruritus (*n* = 2039, ROR 4.9), urticaria (*n* = 985, ROR 4.98), and angioedema (*n* = 285, ROR 5.22), whereas Loratadine did not show significant related ADE reports. Moreover, both antihistamines showed notable signals in eye disorders but manifested differently. Cetirizine was mainly associated with eye pruritus (*n* = 180, ROR 5.05), while Loratadine was more likely to result in increased lacrimation (*n* = 156, ROR 7.2).

Additionally, this study identified some other high signal ADEs unique to each drug. For Cetirizine, it may induce gastrointestinal disorders, primarily presenting as lip swelling (*n* = 155, ROR 3.71). More severely, Cetirizine may induce pericarditis (*n* = 138, ROR 8.13). On the other hand, Loratadine may lead to the occurrence of respiratory, thoracic, and mediastinal disorders, mainly manifesting as rhinorrhea (*n* = 326, ROR 6.75), sneezing (*n* = 251, ROR 15.24), and nasal congestion (*n* = 185, ROR 4.25) among other nasal symptoms. Trouble arises as Loratadine is associated with the occurrence of nervousness in psychiatric disorders (*n* = 145, ROR 3.3).

### 3.3 Comparison of safety signals in two SOCs

**FIGURE 3:**
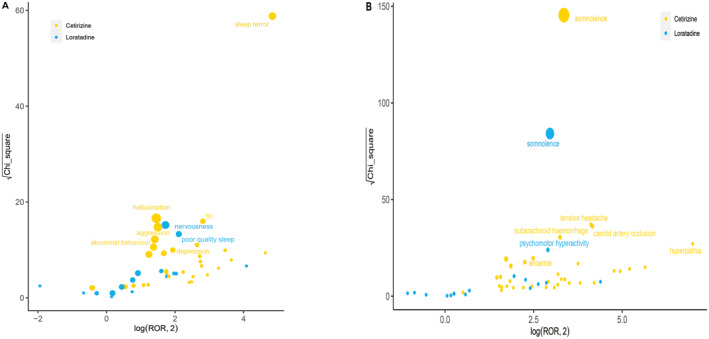
Comparison of nervous and psychiatric system safety signals between Cetirizine and Loratadine. (A) Nervous system disorders; (B) Psychiatric disorders Figure 3(A&B), respectively showed the mining results of ADE signals of Cetirizine and Loratadine in two SOCs. The *X*‐axis was log^2^ROR, and the *Y*‐axis was the square root of the *χ* ^2^ value. All points in the figure represented the mined ADE signals, and the size of points represented the number of ADEs. ROR was used to determine the location of each ADE in the figure. When the position of the point in the graph was higher and further, both algorithms proved that the signal of the ADE was strong. ADEs, adverse drug events; ROR, reporting odds ratio; SOC, system organ class.

We compared the ADE signals in two different SOCs and found distinct characteristics for signals associated with different drugs, as illustrated in Figure 3. In nervous system disorders, somnolence emerged as the strongest signal associated with Cetirizine, as indicated by ROR and chi‐square values. Simultaneously, based on the mined report counts, the strongest signal for Loratadine within the same SOC was also somnolence. Additionally, in psychiatric disorders, sleep terror stood out prominently in Cetirizine‐related ADEs. It is worth noting that more severe adverse reactions such as hallucination, aggression, and abnormal behavior were also strongly associated with Cetirizine. Conversely, the ADEs most associated with Loratadine in psychiatric disorders were nervousness and poor‐quality sleep.

## 4 DISCUSSION

In this study, we conducted a comprehensive analysis and comparison of ADE reports associated with two widely used second‐generation antihistamines, Cetirizine and Loratadine, using the FAERS database. Consistent with previous research evidence,^17^ our findings indicated that patients with Cetirizine or Loratadine related ADE reports had similar ages (average age around 45 years old) with a significantly higher proportion of female reporters (male‐to‐female ratio, cetirizine: 50.80 vs. 26.75%, Loratadine: 53.62 vs. 32.44%). This might be attributed to the higher prevalence of allergic diseases among females and their increased attention to the occurrence of adverse reactions.^18^ Additionally, as the FAERS database is predominantly led by the United States, the majority of ADE reports originated from the United States, particularly for Loratadine (cetirizine: 79.74%, Loratadine: 94.49%), potentially indicating geographical bias. With the surge in allergic diseases, ADE reports related to Cetirizine and Loratadine have increased annually. However, interestingly, 2020 seemed to be a turning point for Loratadine related ADEs with a subsequent decline in related reports, while ADEs related to Cetirizine continued to show a rapid upward trend. This to some extent reflected the current situation where Loratadine's market seemed to be overshadowed by other medications.

Since both Cetirizine and Loratadine are OTC medications, we found that a portion of ADE reports were due to human factors such as incorrect drug use, including use for inappropriate indications or age groups, incorrect dosages and frequencies, and expired medications (cetirizine: *n* = 6542, 23.3%, Loratadine: *n* = 8395, 29.4%). Of note, Loratadine might cause nasal symptoms such as rhinorrhea, sneezing, and nasal congestion, while Cetirizine did not appear to have such effects.

Furthermore, we found that despite being second‐generation antihistamines, Cetirizine and Loratadine still exhibited significant neurological and psychiatric side effects. Among them, the most prominent ADEs associated with Cetirizine were somnolence and disturbance in attention, while for Loratadine, somnolence and nervousness were more predominant. Further exploration revealed a strong correlation between Cetirizine and neurological and psychiatric symptoms such as somnolence and sleep terror. Despite the limited ability of second‐generation antihistamines to penetrate the blood‐brain barrier and their high selectivity for H1 receptors without anticholinergic effects, studies have shown that they still exhibit varying degrees of sedative‐related side effects.^19^ To be specific, studies have indicated that among second‐generation antihistamines, cetirizine, desloratadine, and Loratadine, especially at high doses, might have more sedative effects compared to fexofenadine and levocetirizine, leading to adverse reactions in the nervous and psychiatric systems.^7^

It is worth noting that several studies^20^, ^21^, ^22^ have pointed out that Cetirizine was more effective than Loratadine in clinical treatment responses when comparing with Loratadine. However, it was unreasonable to solely judge the effectiveness of a drug. Our study found that Cetirizine appeared to be more likely than Loratadine to cause psychiatric and neurological‐related ADEs, consistent with previous research evidence. Specifically, an observational study^23^ conducted across nine European countries compared the treatment effects and tolerability of 7274 patients receiving oral antihistamine therapy. The results showed a significantly higher rate of somnolence caused by cetirizine, nearly double that of levocetirizine, fexofenadine, and desloratadine. Tashiro et al.^24^ utilized positron emission tomography to evaluate the sedative intensity of antihistamines and found that single doses of 10 and 20 mg of Cetirizine orally could occupy 12.5% and 25.2% of H1 receptors in the frontal cortex and cingulate gyrus, respectively. Cetirizine could cause significant somnolence, and even lead to more severe neurological and psychiatric adverse reactions such as impaired specific tasks or cognitive impairment, especially when used at doses exceeding the recommended dose (20 mg).^25^, ^26^, ^27^

Cetirizine and loratadine can influence the central nervous system, likely due to their roles as substrates of P‐glycoprotein, which allow for limited blood‐brain barrier penetration. However, cetirizine has a greater capacity to cross this barrier compared to loratadine, as indicated by research showing its higher permeability, which can lead to more pronounced sedative effects.^28^ Furthermore, cetirizine's higher affinity for H1 receptors may result in more significant antihistaminic effects, particularly at elevated doses or with prolonged use, potentially exacerbating adverse reactions.^29^

Some literature indicates that ADEs related to sedation may indeed be associated with particular medication patterns or population characteristics. Carlos et al. noted that antihistamines are associated with an increased risk of emergency visits and hospitalization in elderly patients.^30^ Additionally, Schatz et al. emphasized in their research that there is a significant correlation between drug dosage and adverse reactions, particularly in cases of high doses or polypharmacy, which may exacerbate sedative effects.^31^ Therefore, when using second‐generation antihistamines such as Cetirizine and Loratadine, strict control of dosage and frequency should be implemented, and patients' central nervous system responses should be closely monitored to avoid serious AEs.

On the other hand, it is worth noting that previous studies^32^, ^33^, ^34^, ^35^ have indicated no clinically significant cardiac effects associated with second‐generation antihistamines such as Loratadine, cetirizine, and fexofenadine. However, our study found a significant association between Cetirizine and pericarditis, despite the low incidence rate (*n* = 138, 0.5%, ROR 8.13). Although the incidence of pericarditis is low, its potential severity should not be overlooked. Clinically, physicians prescribing cetirizine should consider this risk, especially in patients with a history of heart disease or other related risk factors. Patients who experience chest pain, difficulty breathing, or other heart‐related symptoms after taking cetirizine should seek medical attention promptly for appropriate evaluation and management.

In conclusion, although this study provided reliable scientific evidence for the safety assessment of Loratadine and Cetirizine related ADEs from various perspectives, there were still certain limitations. Firstly, as FAERS is a spontaneous reporting system, it heavily relies on individual reporting of AEs, leading to incomplete and underreporting of data. Spontaneous reports are often based on individual subjective experiences rather than systematic medical records. This subjectivity can lead to certain AEs being over‐reported or under‐reported, thereby affecting the accuracy and reproducibility of the study results.^36^, ^37^ Secondly, this study primarily relied on spontaneous reports from various individuals, especially a large number from consumers, which may lead to reporting bias. Thirdly, countries and regions with a higher number of reports might have sampling bias. Lastly, the lack of further subgroup analyses in our study has resulted in missing some clinical information. Therefore, future studies should focus on more comprehensive and accurate prospective research to properly evaluate the potential occurrence of AEs and safety risks associated with Cetirizine and Loratadine, providing guidance for the selection of antihistamines.

## 5 CONCLUSION

Overall, this study provided a relatively solid scientific foundation for the safety assessment of Cetirizine and Loratadine using the FAERS database. It is noteworthy that compared to Loratadine, Cetirizine posed a greater potential risk in terms of neurological and psychiatric systems. Additionally, this study revealed previously underemphasized potential cardiac toxicity of cetirizine, although at a relatively low incidence rate, the high signal intensity warranted further attention and exploration. Despite the limitations of the database, these preliminary findings are worthy of attention from relevant healthcare professionals to enhance awareness of potential adverse reactions to antihistamines. This research can aid in making informed decisions regarding antihistamine selection and provide valuable insights for future in‐depth research and safety monitoring efforts.

## AUTHOR CONTRIBUTIONS

**Weili Kong**: Conceptualization; methodology; software; data curation; formal analysis; writing–review & editing. **Yijun Dong**: Conceptualization; writing–original draft; visualization; validation. **Sixi Yi**: Writing–review & editing; data curation. **Wei Mo**: Writing–review & editing; validation. **Hui Yang**: Funding acquisition; writing–review & editing; project administration; supervision.

## CONFLICT OF INTEREST STATEMENT

The authors declare that no conflict of interest exists.

## Supporting information

Table S1

## Data Availability

The data that support the findings of this study are available from the corresponding author upon reasonable request.
