# Supplementary material for: High‐risks drug adverse events associated with Cetirizine and Loratadine for the treatment of allergic diseases: A retrospective pharmacovigilance study based on the FDA adverse event reporting system database
Source: Clin Transl Allergy. 2024 Sep 10;14(9):e12392. doi: 10.1002/clt2.12392 (PMC11387460; doi:10.1002/clt2.12392)
Supplement: Supplementary file 1 — Table S1 [file CLT2-14-e12392-s001.docx]

**Supplementary Table 1.** The number of Cetirizine-related ADEs and Loratadine-related ADEs reported yearly after 2004.

| Year | Cetirizine | Loratadine |
| --- | --- | --- |
| 2004 | 2217 | 2390 |
| 2005 | 2266 | 2809 |
| 2006 | 2065 | 2559 |
| 2007 | 2112 | 2530 |
| 2008 | 2572 | 3112 |
| 2009 | 2782 | 3652 |
| 2010 | 4292 | 5456 |
| 2011 | 4549 | 6107 |
| 2012 | 4638 | 7652 |
| 2013 | 4524 | 9413 |
| 2014 | 5730 | 10835 |
| 2015 | 14506 | 10863 |
| 2016 | 15660 | 13674 |
| 2017 | 14843 | 12940 |
| 2018 | 16499 | 12515 |
| 2019 | 17970 | 13259 |
| 2020 | 19474 | 13825 |
| 2021 | 24006 | 12893 |
| 2022 | 29641 | 10644 |
| 2023 | 36039 | 9341 |
